# To determine the sensitivity, specificity, and diagnostic accuracy of diffusion-weighted MRI in localization of non-palpable undescended testes taking laparoscopic findings as the gold standard: A cross-sectional study from Pakistan

**DOI:** 10.1016/j.amsu.2021.103161

**Published:** 2021-12-18

**Authors:** Kamran Fazal, Shaiq Hussain, Faheemullah Khan, Irfan Ullah, Muhammad Junaid Tahir, Qasim Mehmood, Zohaib Yousaf

**Affiliations:** a Department of Radiology, Aga Khan University Hospital, Karachi, Pakistan; b Department of Radiology, Jinnah Postgraduate Medical Center, Karachi, Pakistan; c Kabir Medical College, Gandhara University, Peshawar, Pakistan; d Lahore General Hospital, Lahore, 54000, Pakistan; e King Edward Medical University, Lahore, Pakistan; f Department of Internal Medicine, Hamad Medical Corporation, Doha, Qatar

**Keywords:** Diagnostic accuracy, Diffusion-weighted MRI, Cryptorchidism, Laparoscopy, Radiology

## Abstract

**Background:**

Cryptorchidism, undescended testes, is a pathological condition that is due to failure of descent of testes in the scrotum. This study was aimed to determine the diagnostic accuracy of diffusion-weighted magnetic resonance imaging (DW-MRI) in localization of undescended testes taking laparoscopic findings as the gold standard.

**Methods:**

A cross-sectional study was conducted in the radiology department of a tertiary care hospital from September 27, 2018 to September 26, 2019. A total of 416 patients were included. Abdomen-pelvic and scrotal ultrasound were performed. Preoperative abdominal and pelvic DW-MRI was performed with a 1.5-T MRI system using a body coil. All study patients underwent laparoscopic exploration. Intra-abdominal atrophic testes were treated with laparoscopic orchiectomy and orchiectomy samples were taken for histopathologic examination. DW-MRI findings were correlated with laparoscopic findings. A 2 x 2 table was used to calculate the sensitivity, specificity, positive predictive value, negative predictive value and diagnostic accuracy of DW-MRI taking laparoscopy as the gold standard.

**Results:**

The mean age was 17.08 ± 7.99 years and the mean BMI was 19.36 ± 4.96 kg/m^2^. In our study, 34.1% of cases were diagnosed as undescended testes localized by DWI-MRI and 51% of cases were diagnosed as undescended testes localized via laparoscopy. Sensitivity, specificity, PPV, NPV and accuracy were 65.1%, 98%, 97.2%, 73% and 81.3% respectively.

**Conclusion:**

DW-MRI improves the detection of undescended testes. DW-MRI can be a recommended imaging tool to increase the preoperative diagnostic accuracy of MRI in localizing nonpalpable undescended testes.

## Highlights

• Undescended testis or cryptorchidism is the most common congenital genitourinary anomaly in boys.
• This study was aimed to determine the DW-MRI in localization of undescended testes taking laparoscopic findings as the gold standard.
• DW-MRI improves the detection of undescended testes.
• DW-MRI can be a recommended imaging tool to increase the preoperative diagnostic accuracy of MRI in localizing nonpalpable undescended testes.

## 1 Introduction

Cryptorchidism, generally synonymous with undescended testes, is a pathological condition that is due to failure of the descent of the testis in the scrotum [1]. The most frequent defect of the male urogenital tract at birth is cryptorchidism which causes primitive testicular pathology responsible for infertility [2], and also shows a positive relationship with germ cell tumors. It's ideal management is surgery and the best age for the surgery is < 1 year [3].

Undescended testis or cryptorchidism is the most common congenital genitourinary anomaly in boys and has an incidence of 1–3% in full-term and 15–30% in premature male infants. The etiology of cryptorchidism is not well understood. The undescended testicle may be palpable or nonpalpable; approximately 20% of undescended testes are nonpalpable and either located in the abdomen or the canaliculi or are atrophic or completely absent [4].

Cryptorchidism is associated with impaired fertility, inguinal hernia, and increased risk of testicular cancer [5]. The main reasons for the treatment of cryptorchidism include increased risk of infertility, testicular malignancy, torsion, and/or associated inguinal hernia [6]. Preoperative detection and localization of testes can determine the optimal type of procedure and allow for appropriate future planning. In the case of vanishing or absent testes, imaging findings could obviate the need for surgical exploration [7].

Ultrasound has been the most widely used imaging modality for cryptorchidism. This imaging modality has shown variable diagnostic accuracy in detecting non-palpable testis. It has got poor diagnostic accuracy, necessitating further evaluation with MRI and diagnostic laparoscopy. Diagnostic laparoscopy was found to be a gold standard with 100% sensitivity and specificity for localizing nonpalpable testes and allows for concurrent surgical correction [8].

Use of diffusion-weighted imaging (DWI) with a high *b* value yields information that complements conventional magnetic resonance imaging (MRI) findings, improves identification and localization of undescended testes. It is recommended to use DWI in addition to conventional MRI to increase its preoperative sensitivity and accuracy in localizing nonpalpable testes [9]. Diffusion-weighted MRI (DW-MRI) has been established as a useful diagnostic tool in urogenital imaging as it facilitates the characterization of tissue at the microscopic level [9].

The degree of restriction of water diffusion in biologic tissue is inversely related to tissue cellularity and the integrity of cell membranes. On DWI, the testes have high signal intensity due to their high cell density [10].

A few recently published reports have addressed the diagnostic performance of DWI in the evaluation of various scrotal pathologies such as detection and localization of nonpalpable testes, diagnosis of testicular torsion, and differentiation between benign and malignant pathologies [11].

Proper diagnosis and localization of undescended testis are essential for appropriate management which may include watchful waiting, hormonal treatment, or surgery. Accurate presurgical diagnosis of an absent testis would avoid unnecessary surgery, and correct localization of a testis would limit the extent of surgery and reduce the duration of anesthesia. After a review of the literature, there was a low difference in sensitivity but a much high difference in specificity and accuracy. Hence, this study was aimed to determine the diagnostic accuracy of DWI in localization of undescended testes (cryptorchidism) taking laparoscopic findings as the gold standard in our local population.

## 2 Methods

### 2.1 Participants and study design

The study was conducted in the department of radiology, Jinnah Postgraduate Medical Center, Karachi, Pakistan from September 27, 2018 to September 26, 2019. Non-probability consecutive sampling technique was used for the study. The inclusion criteria was; (i) being male, (ii) age 15 months to 30 years, (iii) patients referred for MRI evaluation for clinical diagnosis, and (iv) patients with empty scrotum with testis not palpable on physical examination. Those patients who were excluded from the study included (i) female patients, (ii) patients with ambiguous genitalia or disorders of sexual development, (iii) patients with previous scrotal surgery, anorectal malformation, and any renal malformation, and (iv) those not giving consent. This work has been reported in line with the STROCSS criteria [12].

### 2.2 Study procedure

This study was approved by the ethical review committee of Jinnah Postgraduate Medical Center (Reference no: JPMC/ERC/086). Patients who were referred to the radiology department of Jinnah Postgraduate Medical Center and fulfilled the inclusion criteria were included in the study. Informed consent was taken before the enrolment of patients and the study was carried out in accordance with the Helsinki Declaration. In the study, Abdomen-pelvic and scrotal ultrasounds were performed for the non-palpable undescended testes. Preoperative abdominal and pelvic DW-MRI examinations were performed with a 1.5-T MRI system (Achiva, Philips Medical Systems, Koninklijke Philips Electronics N.V) using a body coil.

Children below 5 years were well sedated with chloral hydrate and well immobilized during the examination. DWI was performed with *b* values of 50, 400, 800 s/mm^2^. Images were acquired with the patient in supine position, head pointing to the magnet (head first supine; HFS). The body coil was securely tightened using straps to prevent respiratory artifacts. At the center, the laser beam localizer was placed over the symphysis pubis. Chloral hydrate syrup at a dose of 0.5 ml/kg body weight was used for children (less than 5 years) for sedation prior to MRI examination. All study patients underwent laparoscopic exploration under general anesthesia within 2 weeks of the MRI to determine the location of nonpalpable testes. Intraabdominal atrophic testes were treated with laparoscopic orchiectomy. Orchiectomy samples were taken for histopathologic examination. Each DW- MRI report was reviewed by a consultant radiologist having at least 5 years of experience. The reviewer recorded the presence or absence of the undescended testes. DW-MRI findings were correlated with laparoscopic findings (taking as gold standard). All data was recorded by the principal investigator on a predesigned proforma. Bias and confounder were controlled by strictly following the inclusion criteria.

### 2.3 Statistical analysis

Data was entered and analyzed by using the statistical package for social science (SPSS) version 21. Mean ± SD was calculated for quantitative variables and frequency and percentages were calculated for qualitative variables. A 2 x 2 table was used to calculate the sensitivity, specificity, positive predictive value, negative predictive value, and diagnostic accuracy of DW-MRI taking laparoscopy as the gold standard. Effect modifiers were controlled through stratification of age and location. Post-stratification 2 x 2 table was used to calculate sensitivity, specificity, PPV, NPV and diagnostic accuracy.

## 3 Results

Total of 416 male patients with age between 6 months and 30 years, meeting the inclusion criteria were included in the study. The mean age was 17.08 ± 7.99 years. The mean height, weight, and body mass index (BMI) were 136.62 ± 24.60 cm, 38.63 ± 17.28 kg and 19.36 ± 4.96 kg/m^2^ respectively. In our study, 34.1% of cases were diagnosed as undescended testes localized by DW-MRI. Fifty one percent of cases were diagnosed as undescended testes on laparoscopy (Table 1). The age and BMI categories are stratified according to localization of undescended testes by DW-MRI and laparoscopy using chi sequare test (Table 2).

**Table 2:** Stratification of age and BMI groups according to diagnosis of undescended testes by DW-MRI and Laparoscopy.

Parameters	DW-MRI	Laparoscopy			P-value
		Yes	No	Total	
Overall	Yes	138 (97.2)	4 (2.8)	142	0.000^a^
	No	74 (27.0)	200 (73.0)	274	
	Total	212	204	416	
Age <18 years	Yes	67 (94.4)	4 (5.6)	71	0.000^a^
	No	40 (29.6)	95 (70.4)	135	
	Total	107	99	206	
Age ≥18 years	Yes	71 (100.0)	0 (0.0)	71	0.000^a^
	No	34 (24.5)	105 (75.5)	139	
	Total	105	105	210	
Non obese	Yes	134 (97.1)	4 (2.9)	138	0.000^a^
	No	72 (27.2)	193(72.8)	265	
	Total	206	197	403	
Obese	Yes	4 (100.0)	0 (0.0)	4	0.009^a^
	No	2 (22.2)	7 (77.8)	9	
	Total	6	7	13	

a P-Value ≤0.05 considered as significant.

Sensitivity, specificity, positive and negative predictive values, and diagnostic accuracy of DW- MRI were calculated for localization of undescended testes taking laparoscopy as gold standard. The results are shown in Table 3.

**Table 1:** Descriptive Statistics of patients.

Variables		Mean (±S.D)	Frequency (%)
Age (years)		17.08 ± 7.99	
	<18 years	10.06 ± 4.27	206 (49.52)
	≥18 years	23.96 ± 3.60	210 (50.48)
Height (cm)		136.62 ± 24.60	
Weight (Kg)		38.63 ± 17.28	
BMI (Kg/m^2^)		19.36 ± 4.96	
	Non-Obese (<30 kg/m^2^)	18.89 ± 4.26	403 (96.88)
	Obese (≥30 kg/m^2^)	34.08 ± 1.58	13 (3.12)
Undescended testes by laparoscopy	Yes		212 (51.0)
	No		204 (49.0)
Undescended testes by DW-MRI	Yes		142 (34.1)
	No		274 (65.9)

**Table 3:** Diagnostic accuracy of DW-MRI in localization of undescended testes taking laparoscopy as gold standard.

Parameters	Sensitivity	Specificity	PPV	NPV	Accuracy
Overall	65.1%	98%	97.2%	73%	81.3%
Age <18 years	62.6%	96%	94.4%	70.4%	78.6%
Age ≥18 years	67.6%	100%	100%	75.5%	83.8%
Non obese	65%	98%	97.1%	72.8%	81.1%
Obese	66.7%	84.6%	100%	77.8%	84.6%

## 4 Discussion

Preoperative awareness of the testicular position in cases of the non-palpable undescended testis is valuable for surgical planning, facilitating the placement of the surgical incision, as well as the choice of operative technique, especially when performing laparoscopic orchidopexy and in the first step of the Fowler–Stephen's maneuver in cases of intra-abdominal gonads [13].

Hypertrophic contralateral testis could be correlated with absent or atrophic testis [14,15]. Nevertheless, this does not preclude surgical exploration of the abdomen and/or inguinal canal, since the sign of compensatory hypertrophy is not specific enough [16]. If the testis is impalpable, an ultrasound examination is always performed. In expert hands, ultrasound has a high positive predictive value for inguinal located testes, such as 91% with sensitivity of 78% [17].

When ultrasound is done by a pediatric radiologist, and testis is located in an inguinal location, a primary inguinal exploration can be considered, preventing an unnecessary diagnostic laparoscopy [18]. Laparoscopy has become the new “gold standard” diagnostic method for impalpable testis. In addition to being a diagnostic method, it has therapeutic advantage [14].

The overall mean age of the patients included in our study was 17.08 ± 7.99 years which is in contrast to the mean age calculated by Zainab et al. as 7.21 ± 1.43 years [1]. Age at diagnosis is an important prognostic factor in the case of undescended testes because the risk of developing testicular cancer and infertility increases with age.

An important step in the surgery is a thorough clinical examination once the boy is under general anesthesia, as a previously impalpable testis might be identified during this examination and subsequently, the planned laparoscopic surgical approach can be changed to standard inguinal orchidopexy. However, even under anesthesia, it can be difficult to palpate a testicular nubbin in the scrotum, especially in an obese child [18]. Some studies show that although a nonpalpable testis was diagnosed by a pediatric surgeon and urologist, an inguinal testis was found in 21–85% of the patients during surgery [19].

The standard management of impalpable testes is surgical, which could be challenging and controversial. Orchidopexy is indicated if the testis is not located in the scrotum after 6 months as it improves fertility rates and allows close monitoring for the development of possible testicular masses [20].

Chan et al. performed surgery early enough, but they also wait a little bit longer compared to the timing of treatment for patients with palpable undescended testis (18, instead of 12 months), for the scrotal sac to allow them to place a testicular prosthesis. According to the study treatment protocol, they started with the laparoscopic examination which reported the easiest and most accurate way to locate an intraabdominal testis [9,21].

Subsequent testicular nubbin removal or orchidolysis and orchidopexy can be carried out using the same approach to achieve the therapeutic aims [21]. Some surgeons tend to start with inguinal surgical exploration, with possible laparoscopy during the procedure [22]. After years of practice, it might be advisable to consider an inguinal approach, together with examination under anesthesia as the first line of treatment. In that way, laparoscopies might be unnecessary and time-consuming, especially in obese children [9].

Another controversial issue is the need for the removal of testicular nubbin. Nubbin is a congenital condition in which no normal testicular tissue can be identified following exploration for a clinically impalpable testis [23]. This includes the presence of a testicular remnant, nodule, or strand of testicular/paratesticular tissue at the end of the spermatic cord. The concern with the presence of seminiferous tubules is that there is the potential for viable germ cells to be present, although they may have not been identified specifically on histological analysis. This variance may be secondary to the different histopathological analysis practices in the various studies. There is only one case of intratubular germ cell neoplasia in a testicular remnant reported in the literature and this was not immunohistochemically supported [24]. The absence of a testis from the scrotal sac represents a psychologically traumatic experience in males of any age from childhood to the elderly [25,26].

In our study, 34.1% of cases were diagnosed as undescended testes localization by DW-MRI. As far as laparoscopy is concerned, 51% of cases were diagnosed as undescended testes localization. This is in contrast to the study findings of a research conducted by Zainab et al. in which DW-MRI detected cryptorchidism in 75.60% of the patients [1].

In our study, almost 33% of the patients were true positive, correctly diagnosed and 48% of the patients were true negative, correctly diagnosed. Sensitivity, specificity, PPV, NPV and accuracy were 65.1%, 98%, 97.2%, 73%, and 81.3% respectively according to our study findings. Many previous studies have calculated the specificity, sensitivity, and diagnostic accuracy of DW-MRI in detecting cryptorchidism. Zainab et al. has shown overall sensitivity, specificity, and diagnostic accuracy of DW-MRI in detecting cryptorchidism as 93.51%, 84.35%, and 91.40% respectively [1]. Abd-ElGawad et al. used conventional MRI and reported sensitivity, specificity, and accuracy of 87%, 50%, and 83% [4]. Using DWI he reported sensitivity, specificity, and accuracy of 91.5%, 66.7%, and 88.67% respectively. In combined use of both conventional and DWI similar study reported sensitivity, specificity, and accuracy of 95.8%, 100%, and 96.2%. False-negative cases were proved by laparoscopy to be atrophic testes especially in patients with older age [4].

### 4.1 Study limitations

The main limitation of our study was the small sample size. Other limitations of the present study include a single-center experience and a nonrandomized study design. It was conducted with urban environment therefore; the results might not be generalizable to larger populations.

## 5 Conclusion

It can be concluded that DWI-MRI improves detection of undescended testes with 65.1% sensitivity, 98.0% specificity and 81.3% accuracy. Diffusion weighted imaging greatly improves the utility of MRI in localization of nonpalpable undescended testes.

## Ethical approval

This study was approved by the ethical review committee of Jinnah Postgraduate Medical Center (Reference no: JPMC/ERC/086).

## Sources of funding

None.

## Author contribution

KF, SH: Study concept or design. KF, SH & FK: Data collection. KF, SH, FK & IU: Data analysis or interpretation. IU, MJT and QM: Writing the paper. ZY: Critical revision of the article.

## Consent

Taken.

## Registration of research studies

Name of the registry: Ethical review committee of Jinnah Postgraduate Medical Center, Pakistan
Unique Identifying number or registration ID: JPMC/ERC/086
Hyperlink to your specific registration (must be publicly accessible and will be checked):

## Guarantor

Zohaib Yousaf (MBBS, MSc, FACP)Department of Internal Medicine, Hamad Medical Corporation, Doha, QatarEmail: zohaib.yousaf@gmail.com.

Irfan Ullah (MBBS)Kabir Medical College, Gandhara University, Peshawar, PakistanIrfanullahecp2@gmail.com+923340968239.

## Data availability

Data will be made available on request.

## Provenance and peer review

Not commissioned, externally peer-reviewed.

## Declaration of competing interest

None.
